# The effect of physical activity on cognition relative to APOE genotype (PAAD-2): study protocol for a phase II randomized control trial

**DOI:** 10.1186/s12883-020-01732-1

**Published:** 2020-06-06

**Authors:** Kyoung Shin Park, Alexis B. Ganesh, Nathaniel T. Berry, Yashonda P. Mobley, William B. Karper, Jeffrey D. Labban, Christopher N. Wahlheim, Tomika M. Williams, Laurie Wideman, Jennifer L. Etnier

**Affiliations:** 1 Department of Kinesiology, University of North Carolina at Greensboro, Greensboro, NC 27402 USA; 2 Under Armour, Baltimore, MD 21209 USA; 3 Department of Psychology, University of North Carolina at Greensboro, Greensboro, NC 27402 USA; 4 Department of Advanced Nursing Practice and Education, East Carolina University, Greenville, NC 27858 USA

**Keywords:** Alzheimer’s disease, Apolipoprotein, BDNF, Dementia, Episodic memory, Executive function, Fluid intelligence, Exercise intervention, MRI

## Abstract

**Background:**

By 2050, the prevalence of Alzheimer’s disease (AD) in the United States is predicted to reach 13.8 million. Despite worldwide research efforts, a cure for AD has not been identified. Thus, it is critical to identify preventive strategies that can reduce the risk of or delay the onset of AD. Physical activity (PA) has potential in this regard. This randomized clinical trial aims to (a) test the causal relationship between PA and AD-associated cognitive function for persons with a family history of AD (FH+), (b) determine the moderating role of apolipoprotein epsilon 4 (*APOE4*) carrier status on cognition, and (c) assess cerebral structure, cerebral function, and putative biomarkers as mediators of the effects of PA on cognition.

**Methods:**

We are recruiting cognitively normal, middle aged (40–65 years) sedentary adults with FH+. Participants are randomly assigned to a 12-month PA intervention for 3 days/week or to a control group maintaining their normal lifestyle. Saliva samples are taken at pre-test to determine *APOE* genotype. At pre-, mid-, and post-tests, participants complete a series of cognitive tests to assess information-processing speed, verbal and visual episodic memory, constructional praxis, mnemonic discrimination, and higher-order executive functions. At pre- and post-tests, brain imaging and blood biomarkers are assessed.

**Discussion:**

We hypothesize that 1) the PA group will demonstrate improved cognition compared with controls; 2) PA-derived cognitive changes will be moderated by *APOE4* status; and 3) PA-induced changes in neural and blood biomarkers will contribute to cognitive changes and differ as a function of *APOE4* status. Our results may provide important insights into the potential of PA to preserve neurocognitive function in people with a heightened risk of AD due to FH+ and as moderated by *APOE4* status. By using sophisticated analytic techniques to assess *APOE* as a moderator and neurobiological mechanisms as mediators across trajectories of cognitive change in response to PA, we will advance our understanding of the potential of PA in protecting against AD.

**Trial registration:**

ClinicalTrials.gov NCT03876314. Registered March 15, 2019.

## Background

### Background and rationale

Dementia is a general term describing a group of symptoms associated with memory decline and other cognitive impairments severe enough to disturb a person’s ability to perform daily activities. Alzheimer’s disease (AD) is the most common form of dementia (approximately 60–80%) and is a progressive, neurodegenerative ailment [1]. The global prevalence of AD is expected to reach 152 million by the year 2050 and the global costs of dementia equated to $1 trillion in 2019, and these costs are predicted to reach $2 trillion by 2030 [2]. Although scientists are exploring disease-modifying pharmacological interventions [3], there is currently no known cure for AD [4, 5]. Therefore, researchers are also focusing on interventions with the potential to delay the onset of the disease by targeting modifiable risk factors [6]. Delaying the onset of AD by 1 year could reduce its incidence by 11% [7] and delaying the onset by 5 years could reduce the number of Americans with AD by 29–43% and total health care costs by $83 - $367 billion [1].

### The potential of physical activity

About one-third of AD cases worldwide are related to modifiable risk factors, with the largest proportion of cases in the United States attributable to a lack of physical activity (PA) [5]. Retrospective [8, 9] and prospective [10–13] studies have shown that greater PA predicts better cognitive performance and a decreased risk of dementia. Meta-analytic reviews of this literature have reported promising benefits of PA specifically for the prevention of AD (OR = 0.14–0.72) [10, 14–17]. Furthermore, experimental studies have shown that increasing PA results in improved cognitive performance in cognitively normal adults [18–21], people with mild cognitive impairment (MCI) [22], and persons with self-reported memory complaints [23]. Meta-analytic reviews have reported a moderate degree of beneficial effects of PA on cognitive function in cognitively normal older adults [24–27]. Therefore, it is important to further our understanding of how PA might be used to prevent or delay AD.

Individuals with a family history of AD (FH+) and who are carriers of the apolipoprotein epsilon 4 allele (*APOE4*+) have a 46–61% heightened risk of AD, making them an important target for prevention [28]. Importantly, *APOE4* contributes independently to the risk of AD, above and beyond FH [28–30]. Cross-sectional and prospective studies have shown that the relationship between PA and cognition is moderated by *APOE4* such that larger cognitive benefits are typically [31–34], but not always [35, 36], reported for *APOE4*+ as compared to *APOE4* non-carriers (*APOE4*-). In cognitively normal older women with a FH+, the relationship between aerobic fitness and cognitive performance was more positive for *APOE4*+ than for *APOE4*- [37]. However, recently it was reported that participation in an 8-month PA program improved cognition in individuals with a FH+ irrespective of *APOE4* carrier status [38]. To date, there is a critical gap in the literature because there is no evidence from randomized control trials (RCTs) regarding the extent to which PA affects cognition in cognitively normal, middle-aged, FH+ individuals relative to *APOE4* carrier status.

### Lack of comprehensive neurocognitive assessment

The extant literature also lacks a comprehensive assessment of AD-sensitive cognitive changes in response to PA. Every area of cognitive function is distinctively affected by AD [39] and thus needs to be appraised in terms of its responsiveness to PA. Well-established patterns of cognitive deterioration in AD appear in verbal and visual episodic memory, auditory and visuospatial information processing, visuospatial constructional praxis, attention, and executive functions (EFs) including working memory, set-shifting, inhibitory control, planning, and fluid intelligence [39]. RCTs on the benefits of PA interventions for cognition in people with a heightened risk for AD (i.e., MCI) have focused on measures of general cognition [23], a subset of the domains of EF [22] or memory [40, 41]. The use of these limited measures impedes our ability to determine how specific aspects of cognitive function differentially respond to PA [23]. Therefore, a comprehensive assessment is needed to fully determine the effects of PA on various aspects of cognition for individuals with a heightened risk of AD.

In addition to assessing behavioral measures of cognition, we will also assess changes in blood biomarkers and brain health in individuals with FH+ relative to their *APOE4* status and the PA intervention. With regard to blood biomarkers, we are focused on putative biological markers sensitive to effects of PA and/or that have implications for AD. Brain-derived neurotrophic factor (BDNF) is important for neuronal survival, growth, and maintenance [42] and has been implicated in the consolidation of memory [43–45]. Peripheral levels of BDNF decrease during AD, and higher BDNF is associated with slower decline in AD [46]. Although evidence is inconsistent, chronic PA increases peripheral BDNF [22, 47, 48] and exercise-induced changes in peripheral BDNF are associated with hippocampal volume changes [21]. This evidence supports BDNF as a potential mechanism which could mediate the benefits of PA for cognition. In this study, BDNF is of primary interest, but we assess additional biomarkers implicated in the relationship between PA and AD (see Table 1 for the summary of rationale supporting the inclusion of each biomarker). Changes in biological markers in response to PA will also be examined relative to *APOE4* carrier status.

**Table 1:** Biomarkers to be measured and potential mechanisms

Biomarker	Potential mechanism or proposed pathway
BDNF	Crucial role in plasticity of central and peripheral nervous systems [49]; Exercise responsive [50, 51]; Binds to tropomyosin-related kinase B (TrkB) receptor, ↑ glucose uptake, interacts with autonomic nervous system [42]
SAP	Biomarker of progression to AD [52, 53]
Albumin	Neuroprotective due in part to anti-oxidative properties [53, 54]
Glucose	Levels altered by exercise and related to irisin and/or BDNF; high levels have negative effect on neuroplasticity [55, 56]
IGF-I	Interacts with BDNF in response to exercise; neural plasticity [57–59]
APOE	Linked to Aβ clearance and lipid homeostasis [53, 60]
alpha-2 macroglobulin	Proteinase inhibitor associated with AD disease severity [53, 61]
Insulin	Altered by exercise and irisin; ↑ insulin sensitivity [62, 63]
Irisin	Exercise ↑ FNDC5/irisin release from muscle; ↑ BDNF transcription in hippocampus [42, 64, 65]
TNF-α	Pro-inflammatory [66, 67]; related to dementia [68, 69]

*AD* Alzheimer’s disease, *APOE* apolipoprotein epsilon 4 allele, *BDNF* Brain-derived neurotrophic factor, *CRP* C-reactive protein, *FNDC5* fibronectin type III domain containing 5, *IGF* insulin-like growth factor, *IL* interleukin, *SAP* serum amyloid P, *TNF* Tumor necrosis factor

Using magnetic resonance imaging (MRI), we will also assess brain structure volume, task-evoked brain activity, functional connectivity, white matter microstructure, and white matter hyperintensities as measures of brain health. Non-experimental studies have shown that greater aerobic fitness is associated with greater hippocampal volume [70–72] and less age-related neural degeneration, particularly in the prefrontal, superior parietal, and temporal cortices [73]. Baseline PA is predictive of hippocampal volume 9 years later [74] and aerobic fitness is associated with less age-related dysfunction in functional connectivity [75]. In RCTs, PA interventions have resulted in increased grey matter in the prefrontal cortex [76], hippocampal volume [21], resting cerebral blood flow [77], task-evoked activity [78], white matter integrity [79], and functional connectivity in cognitively-relevant regions [80, 81].

Brain health differs by *APOE4* status in cognitively normal adults, evidenced by right hippocampal atrophy [82, 83], decreased cortical thickness [84, 85], and reduced grey matter [86, 87] in *APOE4*+ compared to *APOE4*- individuals. White matter deficits, including decreased diffusion anisotropy [88–91], have also been found in *APOE4*+, with the lowest fractional anisotropy in individuals who are FH+ and *APOE4*+ [92]. Further, differences in functional connectivity as a function of *APOE4* status have been shown in young [93], middle-aged, and older adults [94, 95]. However, to date, few studies have reported the moderating effects of *APOE* on the relationship between PA and brain health, and results have been inconsistent. In one study, PA was found to be associated with greater memory-related brain activation, with the strongest associations in people with *APOE4*+ [96]; however, another study found a positive association between PA and hippocampal volume that was not moderated by *APOE4* status [97]. To our knowledge, there is no evidence from RCTs regarding the effects of a PA intervention on brain health relative to *APOE4*. In this study, we will assess changes in brain health in response to a PA program and explore the moderating role of *APOE4* carrier status.

### Study objectives

This protocol describes the design and methods of a RCT aiming to 1) test the causal link between PA and cognitive performance in persons with a FH+, 2) determine if the effect of PA on cognitive performance is moderated by *APOE4* carrier status, 3) assess the extent to which measures of brain health and putative biomarkers serve as mediators of the effects of PA on cognition and 4) determine the extent to which these mediated relationships are moderated by *APOE4* carrier status. Our results might provide important insights into the potential of PA to foster cognitive performance in those with a heightened familial and genetic risk of AD. Given that midlife PA has been shown to reduce the risk of AD [98, 99], beneficial responses to PA could delay AD in this particularly susceptible population, and thereby meaningfully reduce the prevalence of AD [6].

## Methods and design

### Study design and setting

In this RCT, middle-aged sedentary adults with a FH+ are pre-tested (baseline), randomly assigned to a 12-month PA intervention or a usual-care control, mid-tested (6 months after baseline), and post-tested (12 months after baseline). The study sites are laboratories at the Department of Kinesiology and the Gateway MRI Center at the University of North Carolina at Greensboro located in Greensboro, North Carolina, USA, along with local YMCAs where the PA intervention is provided. This study was approved by the university’s Office of Research Integrity and this protocol is reported in accordance with the SPIRIT guidelines [100]. See the SPIRIT checklist in additional file 1 for more information.

### Participants

We plan to recruit 240 middle-aged (40–65 years), cognitively normal adults with a FH+ who are currently participating in moderate to vigorous intensity exercise fewer than 3 days/week for 30 min or more. Unlike ongoing PA trials with older adults (> 65 years), this study is uniquely positioned to address key gaps in knowledge by focusing on cognitively normal middle-aged adults with a heightened risk for AD (FH+, *APOE4*+). These are people for whom benefits to cognitive performance and to underlying neurological and biological mechanisms may be more readily observed, and for whom effective interventions may ultimately delay cognitive decline. Therefore, we adopt an early therapeutic strategy that may offer the best opportunity for protective effects [101–104] by focusing on middle-aged adults. Importantly, the use of standardized measures allows us to discuss our results in the context of other studies, especially a similar clinical trial regarding the effects of physical activity on cognition and brain health in 65- to 80-year-old cognitively normal adults (Investigating Gains in Neurocognition in an Intervention Trial of Exercise, IGNITE trial; [105]).

#### Eligibility criteria

The goal of the eligibility criteria is to include middle-aged English-speaking adults (40–65 years) with FH+ who are cognitively normal, who are not otherwise clinically impaired, who are healthy enough for exercise, and who are identified as sedentary according to American College of Sports Medicine (ACSM)'s guidelines [106]. Sedentary is defined as participating in physical activity at a moderate to vigorous intensity for 30 min or more, fewer than 3x/week over the last 3 months and is assessed by self-report.

The FH of dementia is defined as one first-degree or 2 second-degree relatives diagnosed with non-specific dementia or AD. The relatively broad inclusion criteria with respect to FH reflect that 1) the majority (60–80%) of individuals diagnosed with non-specific dementia have AD and 2) volunteers at the lower end of the inclusion age range (e.g., 40–45 years) may have parents who are not sufficiently old to be likely to be diagnosed with AD. Individuals who only reported family members with diagnosed forms of dementia other than AD, such as vascular dementia or dementia with Lewy bodies, are not included. Although *APOE4*+ make up approximately 24% of the general U.S. population < 65 years of age [107], based upon previous research [37, 108], recruiting adults with a FH+ increases the percent of *APOE4*+ to ~ 35%. Thus, by recruiting 240 people with a FH+, we anticipate successfully enrolling approximately 80 *APOE4*+ participants. See Table 2 for an overview of inclusion and exclusion criteria.

**Table 2:** Inclusion and exclusion criteria

Inclusion criteria	Instrumentation	Cutoff
40–65 years of age	Telephone interview	
Family history of dementia	Telephone interview, Risk Evaluation and Education for Alzheimer’s disease (REVEAL) questionnaire	1 first degree relative or 2 second degree relatives diagnosed with dementia or AD [109]
Ability to communicate in English	Telephone interview	
Not meeting PA guidelines	American College of Sports Medicine (ACSM)'s guidelines	30 min of moderate intensity PA fewer than 3x/week for the last 3 months [106]
Exclusion criteria	Instrumentation	Cutoff
Potential cognitive impairment	Modified Telephone Interview for Cognitive Status (TICS-m)	Total score < 33 [110]
Potential cognitive impairment	Montreal Cognitive Assessment (MoCA)	Total score < 26 [111]
Current use of medications to treat symptoms of AD or that adversely affect cognition	Self-report to a survey	
Cannot attend PA because of cardiovascular, metabolic, or renal disease, or orthopedic limitations	Medical Health History (MHH), physician clearance	
History of neurologic, psychiatric, or active functionally disabling disease, or any other conditions that might limit exercise or jeopardize participants	MHH questionnaire	
Depression	Center for Epidemiological Studies Depression Scale - Revised (CESD-R)	Total score > 16 and having anhedonia or dysphoria nearly every day for the past 2 weeks, and 2 or more additional symptoms either nearly every day for the past 2 weeks or 5–7 days in the past week or indication for suicidal ideation [112]
Uncorrected hearing or visual impairments	Self-report to a survey	
Plan for traveling for an extended period (more than 1 month) during the course of the study	Self-report to a survey	

#### Recruitment strategies

Participants are being recruited from six counties in North Carolina: rural (Randolph, Rockingham), regional city and suburban (Davidson, Alamance), and urban (Guilford, Forsyth). This recruitment strategy increases the diversity of our sample and contributes to the reproducibility of the results. The 6 counties have a combined population of approximately 469,588 in this age range [113]. We are advertising the study via local television, radio, billboards, car magnets, and newspapers; through emails, newsletters, and flyers distributed to support groups, places of worship, medical facilities, community centers, restaurants, and other locations; by giving community talks; and with social media posts. Given the demographics of the recruitment region, we aim to include approximately 125 women and 115 men.

#### Informed consent

Participants in this study are provided written informed consent at the first, in-person visit at the pre-test by research staff. More specifically, participants are explained all of the procedures, risks, potential benefits, and issues of confidentiality, provisions for collection and use of individual data and biological specimens, and provisions for care in the event of any adverse events. Participants are provided with enough time to make an informed decision, including time to ask any questions and discuss the study with the research staff. All participants in this study must go through a screening test to assess cognitive normality prior to being consented to participate in the study. Therefore, all participants are considered capable of ethically and medically consenting for participation on their own behalf.

### Procedures

Eligibility for participation is initially assessed during a telephone interview. Individuals complete additional screening through online survey sets prior to their initial visit and complete the final assessments to determine eligibility at the pre-test. Eligible participants complete on-site pre-, mid-, and post-intervention assessments. After the pre-test, participants are randomly assigned to a 12-month PA intervention or a usual-care condition. See Table 3 for an overview of our protocol time schedule at five timepoints; screening (T0), pre-test (T1), allocation (T1.5), mid-test (T2), and post-test (T3).

**Table 3:** An overview of the study time schedule for assessments and interventions

Timepoint	T0	T1	T1.5	T2	T3
Screening:					
- Modified Telephone Interview for Cognitive Status (TICS-m)	X				
- MRI safety screening	X				
- Medical Health History (MHH)	X				
- Questions from ACSM guidelines for determining risk of physical activity	X				
- Alzheimer’s family history questionnaire (REVEAL)	X				
- Center for Epidemiological Studies Depression Scale Revised (CESD-R)	X				
- Everyday Cognition Questionnaire (ECOG)	X				
- Community Healthy Activities Model Program for Seniors (CHAMPS)	X				
- Pittsburgh Sleep Quality Index	X				
Enrollment					
- Montreal Cognitive Assessment (MoCA)		X			
- Informed consent		X			
Allocation			X		
Interventions:					
- Physical activity			←———————————→		
- Usual care			←———————————→		
Assessments:					
- Cognitive tests		X		X	X
- Neuroimaging		X			X
- Blood sample collection		X			X
- Resting heart rate assessment		X		X	X
- Submaximal exercise test		X		X	X

#### Telephone interview

Research staff describe the study purpose, procedures, and requirements. Individuals who remain interested answer questions relative to the inclusion criteria and complete the Modified Telephone Interview for Cognitive Status (TICS-m) to ascertain initial eligibility for the study. The TICS-m has acceptable sensitivity and specificity in the detection of dementia [114] and amnestic MCI [110] and does not have the same ceiling constraints as other measures of cognitive impairment [110, 115]. Individuals are excluded if they score below the cutoff point of 33, which was determined to prioritize specificity over sensitivity [110]. These participants are contacted by the gerontological nurse practitioner (TMW) who explains the reason for their exclusion, answers questions, and advises them to contact their personal physician. Eligible participants are then sent the survey sets electronically.

#### Survey sets

After the telephone interview, eligible participants are further contacted by email (occasionally by telephone or postal mailing) to complete the following surveys: MRI safety screening questionnaire, Medical Health History (MHH) and medications list, Risk Evaluation and Education for AD (REVEAL, [109]) to evaluate family history of AD, Everyday Cognition Questionnaire [116] to measure perceived cognitive symptoms, Center for Epidemiological Studies Depression Scale – Revised (CESD-R, [112]), Community Healthy Activities Model Program for Seniors [117] and International Physical Activity Questionnaire (IPAQ, [118]) to measure current PA behavior, Pittsburgh Sleep Quality Index [119], a perceived age questionnaire, and demographics. Prior to completing these surveys, informed consent is obtained for these data collection instruments. At this stage, participants who are excluded due to scores on the CESD-R are contacted by the gerontological nurse practitioner (TMW) who explains the reason for their exclusion, answers questions, and advises them to contact their personal physician.

#### Pre-, mid-, and post-intervention assessment

Most participants are tested over two visits at pre- and post-tests and one visit at mid-test. On the first visit of the pre-test, participants first read and sign an informed consent and complete the Montreal Cognitive Assessment (MoCA) for the final screening of cognitive normality. Individuals scoring below the cutoff point of 26 from the MoCA are excluded. The use of this cutoff results in excellent sensitivity in identifying MCI (90%) and AD (100%) and good specificity (87%) [111]. Participants excluded based upon this criterion are referred to the gerontological nurse practitioner (TMW) and advised to discuss their cognitive performance with their physician.

After completing the MoCA, enrolled participants provide a saliva sample for *APOE* genotyping, have resting heart rate (HR) assessed, provide a fasted blood sample, are offered a light meal, perform cognitive tests, and complete a submaximal exercise test. The first visit takes about 5–5.5 h. On the second visit of the pre-test, participants complete an MRI scan, which takes about 1.5 h. Participants unwilling or unable to perform the MRI scan do not attend the second visit. For participants who need to come on 3 days, cognitive testing is divided into set A and set B (see Table S1 in additional file 2 for more information) across two visits to accommodate their scheduling needs. For the estimation of aerobic fitness, participants complete a submaximal exercise test following the Modified Naughton protocol [106], however the first stage (1.0 mph, 0% grade) is omitted. Changes in aerobic fitness provide an indicant of the physiological responsiveness to the PA intervention. At the mid- and post-tests, participants complete the same protocol except that blood sampling and MRI are only taken at the post-test and the saliva sample is not taken again.

### Interventions

After completing the pre-test, participants are randomly assigned to conditions using a computerized randomization procedure implemented in R 3.6.1 [120]. To the extent possible, groups are matched on county of residence, age (40–52/53–65), race (Caucasian/non-Caucasian), and gender (female/not female). Participants are informed of their group assignment by the project coordinator. Project staff conducting the testing are blinded to group assignment throughout the study. Participants are instructed not to discuss group assignment with any research staff during testing sessions.

#### Physical activity condition (PAC)

The PAC was used in previous research in which improvements in memory were observed in association with the program [38]. The PAC was originally based on meta-analytic evidence [25] indicating that in RCTs the largest effects of PA on cognition in older adults were in programs that include both aerobic and strength training (g = 0.59). Hence, the PAC includes both modes of activity. Subjects are asked to attend 3x/week for 1 year and exercise takes place in a group setting. Each subject is encouraged to walk on their own at a moderate intensity (target HR = 40–59% HR reserve) dependent on resting HR and age [106]. Resting HR is assessed by palpation at approximately 3-week intervals. HR and ratings of perceived exertion (RPE) during exercise are assessed mid-way into walking, and RPE is assessed mid-way into strength training. Initially, walking is 10 min/day; this increases gradually until participants are walking for 30 min/day. For strength training, elastic resistance bands and possibly dumbbells are used. Subjects begin with bands with the least resistance, completing one set of 6–15 repetitions for each of 10–15 exercises. As they can complete 15 repetitions for any given exercise in proper form, they are progressed to the next higher resistance band for that exercise. As necessary, we progress to using two bands simultaneously or to dumbbells to continue to appropriately challenge the participants. In the first weeks of the PAC, more time is needed to teach the participants the exercises and to identify appropriate resistance levels. However, by week 8, strength training is completed in 30 min; this is maintained throughout the intervention. In the event that participants cannot attend group sessions for an extended period of time, they are encouraged to continue to exercise as per the PAC protocol.

This PA program is inexpensive, safe, and suitable for community adult programs and home-based exercise recommendations. The program was implemented at local YMCAs until the COVID-19 pandemic and then through Zoom sessions after spring 2020. Fidelity across groups is ensured by using qualified and experienced Fitness Specialists who are trained to implement the program using the PAAD-2 Exercise Program Manual. Fidelity to the program and consistency of implementation across groups are further ensured by regular (at least once / 3 weeks) visits to each group to observe, evaluate, and provide feedback on sessions. Measures of compliance and adherence are obtained at all exercise sessions; groups are compared monthly to ensure no substantive differences exist. If such differences occur, we determine their causes and make necessary changes.

#### Usual-care control (UCC)

We use a UCC in which we ask participants to maintain their normal health practices (e.g., diet, annual physicals) for 1 year. Because the inclusion criteria require that participants not be regularly active, we anticipate that these individuals will not show consistent increases in PA over the year. To reduce effects from experimenter attention, to minimize attrition, and to assess possible cross-contamination, we provide educational materials (covering health topics, but not PA) to UCC participants biweekly. Once per month, we assess their self-reported PA [121] and once every 3 months we contact them by phone to ask if they have had any life changes relevant to the study. To encourage retention, we provide UCC participants with a short-term YMCA membership after the post-test. When participants are offered an intervention after a UCC waiting period, cross-contamination is low (7.1% of studies) and fewer participants drop out from a UCC than an exercise treatment (4.7% fewer) in trials up to 1 year [121].

### Genotype

Saliva samples are collected using Oragene-500 kits. Genomic deoxyribonucleic acid (DNA) is extracted from saliva samples for single nucleotide polymorphism (SNP) analysis. The SNPs associated with the two amino acid residues (codons 112 and 158) are used to identify participants as *APOE4+* or *APOE4-*. Remaining DNA material is stored indefinitely for future analyses. All staff interacting with participants remain blinded to participants’ *APOE4* carrier status.

### Cognitive assessment

**Fig. 1:**
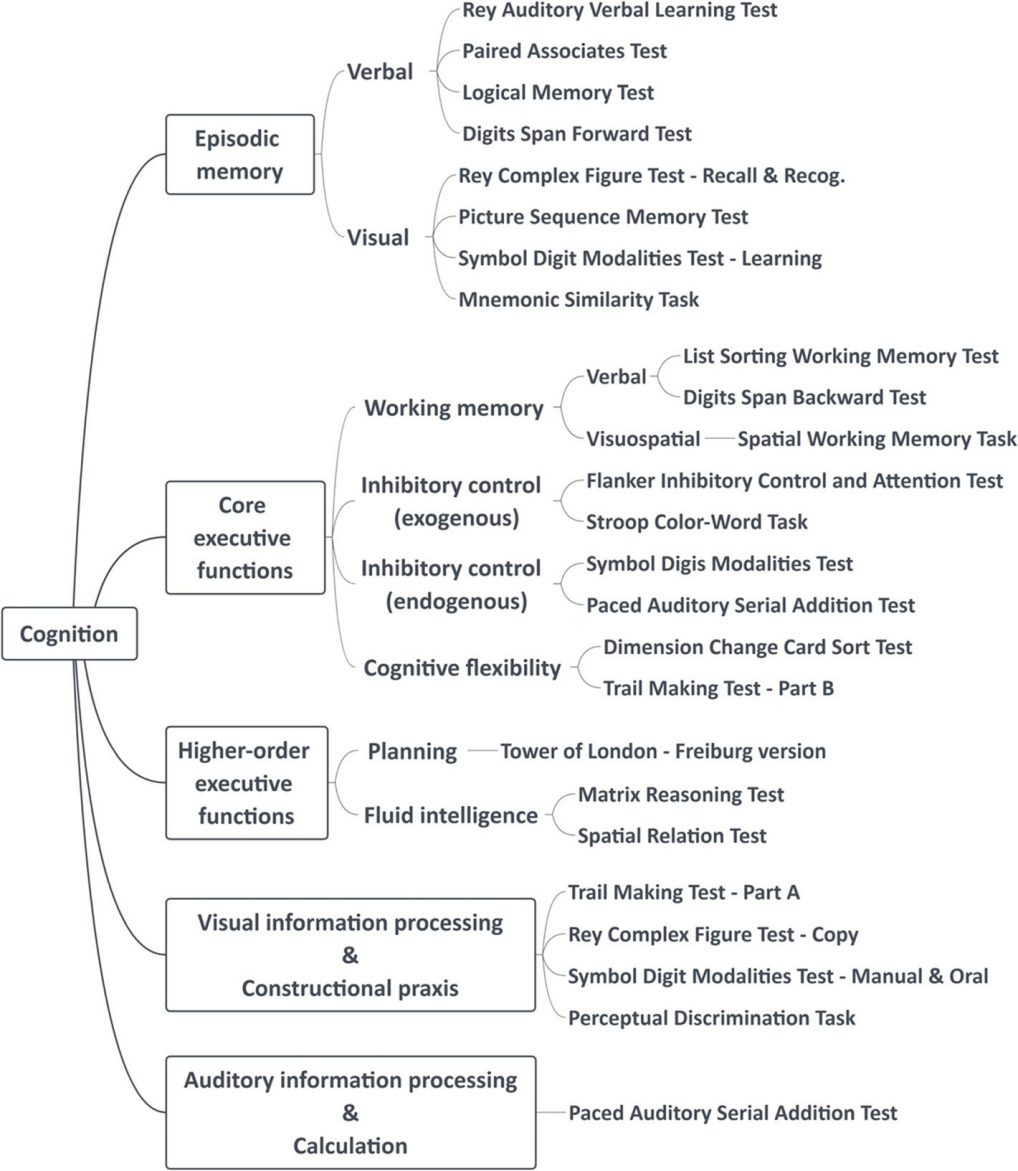
Cognitive domains assessed within PAAD-2 cognitive test battery

We assess cognitive changes based on performance from a custom-built cognitive test battery consisting of well-established cognitive tests. Our primary interest in building the test battery was to include cognitive measures that are sensitive to early-to-advanced stages of AD, especially verbal and visual episodic memory, auditory and visuospatial information processing, visuospatial constructional praxis, attention, and EFs [39]. We organized measures for EFs based on a well-established model [122], which consists of core EFs (inhibitory control, working memory, and cognitive flexibility) and higher-order EFs (planning, reasoning, and problem solving). Specific cognitive tests assessing each domain of episodic memory, core EFs, and higher-order EFs are described in the following section and depicted in Fig. 1. Protocols for each cognitive test are provided in additional file 2. The timeline and order of cognitive tests are described in Table S1 in additional file 2. Our tests are administered using paper/pencil, a desktop computer (Dell, OptiPlex GX110), and/or an iPad 12.0 (Apple Inc.).

#### Episodic memory

We obtain a comprehensive evaluation of verbal and visual episodic memory since memory measures (rate of learning, short-term and long-term memory, retention of information, and retroactive and proactive interference) have been found most sensitive to a PA intervention in previous work [38] and episodic memory – both verbal and visual formats - is most sensitive to early stages of AD [39, 82]. Verbal episodic memory is measured using the Rey Auditory Verbal Learning Test (RAVLT, [123], Digits Span Forward Test [124], and Virginia Cognitive Aging Project (VCAP [125])'s versions of the Paired Associates test [126] and Logical Memory test [127]. Visual episodic memory is measured using the Rey-Osterrieth Complex Figure Test [123, 128], the NIH Toolbox Picture Sequence Memory Test (PSMT [129]), and the Mnemonic Similarity Task (MST [130]). To prevent practice effects from repeated learning of the same stimuli [39], different forms in equivalent difficulties are used for the RAVLT [131, 132], Paired Associates, Logical Memory, PSMT, and MST for the pre-, mid-, and post-tests.

#### Core executive functions

Inhibitory control is defined as including both exogenous (stimulus-driven and involuntary) and endogenous (goal-driven and voluntary) attentional control [122]. Based on this definition, our testing battery includes the NIH Toolbox Flanker Inhibitory Control and Attention Test [133] and the Stroop Color-Word Task (the IGNITE version, [105]) to measure exogenous inhibitory control and the Symbol Digit Modalities Test [134] and the Paced Auditory Serial Addition Test [135] to measure endogenous inhibitory control and selective attention.

Working memory is characterized by active attention, holding information in mind and mentally manipulating or updating it as necessary. This capability is critical for understanding and reacting to stimuli that change over time and requires mental maintenance of what happened earlier so that it can be linked to what comes later [122]. Distinguished by content, two major types of working memory are verbal and nonverbal (visual/spatial) working memory. Our test battery includes the NIH Toolbox List Sorting Working Memory Test [136] and the Digits Span Backward Test [124] to measure verbal working memory, and the IGNITE’s spatial working memory task [21] to measure visuospatial working memory.

Cognitive flexibility, also known as set shifting, mental flexibility, or task switching, involves thinking creatively, seeing things from different perspectives spatially or interpersonally, and quickly and flexibly adapting to changed circumstances and demands [122]. We included the NIH Toolbox Dimensional Change Card Sort Test [133] and the Trail Making Test [137], which additionally assesses attention, visual processing speed, visual search, sequencing, and visuomotor skills.

#### Higher-order executive functions

Planning is defined as a “look-ahead mechanism designed to generate multiple sequences of hypothetical events and their consequences, including the development of stored structured event complexes that can guide movement from an initial to a goal state, execution-linked anticipation of future events, and recognition of goal attainment.” [138], p. 655. Planning is assessed using the Tower of London – Freiburg version [139], a measure of planning with reliable and valid psychometric qualities [140, 141]. Fluid intelligence, also known as the reasoning and problem-solving subcomponents of EFs, represents the ability to do inductive and deductive logical reasoning, problem solving, and to figure out abstract patterns or relations among items [122]. We included the VCAP’s versions of the Matrix Reasoning test [142] and the Spatial Relation test [143] to measure fluid intelligence.

### Blood biomarkers

We use standard protocols for collection and storage of blood samples, assays, and analyses. Whole blood (approximately 45 ml) is collected in EDTA-treated and serum-separating tubes. Approximately 1 ml of whole blood from the EDTA-treated tubes is extracted and mixed at a 1:1 ratio with Halt protease and phosphatase inhibitor (Thermo Fisher Scientific, USA) to protect from protein degradation, which is necessary to assay some of the biomarkers of interest (Table 1). The blood is then centrifuged, and serum, plasma, and Halt-treated plasma are stored in small aliquots at − 80 °C to minimize freeze-thaw issues. Glucose and albumin are analyzed using commercially-available assay kits and requires < 70 μL of serum to run both analytes in duplicate. All other assays are conducted using a multiplex system (Luminex 200S), which uses very small (20–50 μL total) volumes of blood. BDNF is given priority in any insufficient samples. Samples are stored indefinitely.

### MRI

MRI exams are conducted at the Gateway MRI Center at pre- and post-test. Images are acquired on a Tim Trio Siemens 3 T MRI Scanner with a 12 channel receive-only head coil. Sequences collected include a magnetization-prepared rapid acquisition with gradient echo (MPRAGE) T1-weighted structural scan, T2-weighted-Fluid-Attenuated Inversion Recovery (T2-FLAIR) scan, a resting-state echo planar imaging (EPI) scan, famous name discrimination task-evoked EPI scan, T2-weighted scan localized to the hippocampus and a diffusion-weighted scan. Associated images for distortion correction are acquired for EPI and diffusion images with a short spin echo EPI scan in the opposite phase encoding direction. Detailed information for each acquisition protocol is shown in Table 4.

**Table 4:** Magnetic Resonance Imaging Sequences

Sequence	Parameters
T1-weighted MP-RAGE	Resolution = 1.0 × 1.0 × 1.0 mm, TR = 2300 ms, TE = 2.26 ms, TI = 900 ms, FoV = 256 mm, 192 slices
T2-FLAIR	Resolution = 1.0 × 1.0 × 1.0 mm, TR = 5000 ms, TE = 381 ms, TI = 1800 ms, FoV = 256 mm, 192 slices
Resting-state fMRI	Resolution = 3.4 × 3.4 × 3.4 mm, TR = 3000 ms, TE = 30 ms, FoV = 220 mm, 48 slices, EPI factor = 64, 200 measurements
Famous Name Task fMRI (performed twice)	Resolution = 3.4 × 3.4 × 3.4 mm, TR = 3000 ms, TE = 30 ms, FoV = 220 mm, 48 slices, EPI factor = 64, 111 measurements
High resolution Hippocampus	Resolution = 0.4 × 0.4 × 2.0 mm, TR = 8020 ms, TE = 52 ms, FoV = 175 mm aligned perpendicular to the hippocampus, 29 slices
Diffusion weighted acquisition	Resolution = 2.0 × 2.0 × 2.0 mm, TR = 11,000 ms, TE = 97 ms, FoV = 256 mm, 80 slices, 30 diffusion directions

*MP-RAGE* Magnetization Prepared-RApid Gradient Echo, *TR* repetition time, *TE* echo time, *TI* inversion time, *FoV* Field of View, *FLAIR* Fluid-Attenuated Inversion Recovery, *fMRI* functional Magnetic Resonance Imaging

All images are initially examined for quality control and movement artifact immediately following acquisition. The MRI data then get transferred to the server and undergo a rigorous quality control process. The data is converted from dicom to nifti format and the metadata is inspected for sequence accuracy and completeness. The data is then organized into brain imaging data structure (BIDS) format and visually inspected for brain coverage and orientation. Finally, quantitative quality control is completed for each sequence through the MRI Quality Control tool [144].

### Covariates

Because cognitive performance is expected to be associated with age (e.g., [145–147]) and education (e.g., [146, 147]), these variables are included as covariates in the models. Identifying additional individual difference variables that discriminate levels of responsiveness to the intervention may provide important insights for subsequent research. Data on variables that have been identified as important in past research [11, 148–150] are collected and explored as potential covariates. These include sex, blood pressure, smoking, alcohol use, medications and supplements, sleep quality, body mass index, menopausal status, hormone therapy use, diabetes, and cardiovascular risk factors. When assessing FH, detailed information regarding blood-related relatives suspected to have AD or diagnosed with AD is collected. This includes relationship to participant and age at diagnosis. We also collect data on parents who have not been diagnosed with AD (age and health status currently or at time of death). Because a FH+ that is maternal and with a younger age of onset [151] results in higher risk, these variables are included as covariates in statistical analyses. Participants also complete the IPAQ [118] at pre, mid, and post-tests and monthly during the intervention.

### Data analysis

The conceptual model driving our research is inherently a model of change: changes in PA lead to changes in cognitive performance. The current state of the art in statistical models for studying change is latent growth curve (LGC) analysis [152–154] which models trajectories of observed change as reflecting an underlying (“latent”) developmental process. LGC analysis is an ideal statistical tool for testing our conceptual model because it allows us to 1) estimate both mean and individual variation of pretest levels of our outcome variables (e.g., cognitive performance, neurological function/structure, and biomarkers), as well as means and variances of changes in those outcomes as a result of the intervention; 2) test whether PA and *APOE4* status predict trajectories of changes in these outcomes; 3) determine whether changes in cognitive performance are mediated by putative mechanisms; and 4) test whether *APOE4* status moderates the mediational associations.

### Power analysis

We estimated sample size requirements using data from a previous study [38]. Coefficients on which we based our analysis included expected values for slope factors, regression estimates of association between predictors (PA, *APOE4* status) and slope factors, and reasonable estimates of associations of the PA-by-*APOE4* status interaction with slope factors. We conducted Monte Carlo simulations with 5000 replications, requiring estimate and variance bias of < 10%, and coverage > 95% [155], to estimate power for our target sample size of *N* = 240 with attrition rates of 10–30%. Time was coded such that parameter estimates represented expected mean monthly change over the course of the year-long intervention. An initial sample of 240, provided the assumed attrition rates, would have an approximate power of 0.97 to detect a mean slope factor of 0.10, power of 0.86–0.96 to detect parameter estimates of 0.075–0.10 for slope regressed on PA and *APOE4* status, and power of 0.82–0.92 to detect interaction effect estimates of 0.065–0.10 for PA-by-*APOE4* status regressed on slope.

### Data management

Data include hardcopy surveys and data collection sheets, electronic surveys, and results of cognitive testing, MRI scans, genotype, and blood assays recorded electronically. All hard copies of data are linked to subject IDs without names and are stored in a locked cabinet in a laboratory. Electronic data are recorded by IDs and protected by password. A master list linking names to IDs is stored in a password protected file and separate from the data. Saliva and blood samples are stored in a locked laboratory. MRI data are stored on a password protected university server. When data are disseminated, it will not be in a way that would allow for the identification of any individual person. All data are managed using REDCap (Research Electronic Data Capture) hosted at the university [156]. REDCap is a secure, web-based software platform that allows us to control data access at the individual level and apply other rules and constraints that promote data quality.

Pre-test data from the first cohort will be examined to identify data gathering problems to be addressed immediately. The dataset will be cleaned using standard methods to identify impossible and improbable data [157] including frequency distribution checks for outliers and problems in data gathering or entry. Validity checks will be performed as recommended [157]. Standard Operating Procedures (SOPs) for cognitive testing, the PAC intervention, submaximal exercise testing, and blood draws are in place from our Phase I clinical trial (PAAD) [108]. Modifications to the SOP to reflect the addition of cognitive measures and updates to procedures were made prior to participant recruitment. MRI SOPs were developed based upon the existing MRI SOPs from the IGNITE trial [105] to maximize compatibility.

Two independent experts serve on the Data Safety and Monitoring Board to monitor participants’ safety, the progress of the study, and the integrity of data collection. The principal investigator (JLE) makes safety and progress reports twice per year throughout the duration of the study. See additional file 3 (Data Safety Monitoring Plan) for more information. The data obtained in accordance with this protocol will be important to the relevant fields of science, and the PAAD-2 team has established a Data Sharing Plan regarding how, when, what, to whom the data will be disseminated. See additional file 4 for more information.

## Discussion

In this protocol for a phase II randomized clinical trial, we describe the first experimental test of the effects of PA on cognitive performance in cognitively normal, middle-aged adults with a FH+. In addition, we use sophisticated analytic techniques to assess moderated mediation models with *APOE* as a moderator and neurological and biological mechanisms as mediators across trajectories of cognitive change in response to the PA program. Our hypotheses are that 1) in individuals with a heightened risk for AD, PA will improve AD-related cognitive performance relative to controls; 2) the effects on cognitive performance will be moderated by *APOE4* carrier status; and 3) changes in neural and blood biomarker will be observed in response to PA, will differ as a function of *APOE4* carrier status, and will contribute to cognitive changes.

If persons with FH+ who are in the PA program improve more than those in the control group, this will provide causal evidence of the viability of PA as a means of maintaining or improving cognitive performance in middle-age. If there are differential effects relative to *APOE4* carrier status, this would indicate that PA is beneficial for cognition even in those with the greatest familial and genetic risk of AD. Our results are anticipated to elucidate the potential benefit of PA for persons with a FH+ and the differential benefits relative to *APOE4* status. This is a logical next step in advancing our understanding of the potential of PA as a therapeutic intervention for AD. If PA is beneficial for individuals who are FH+ and if PA is particularly advantageous for *APOE4*+, future work could then explore the potential of PA in middle-age to delay, or perhaps prevent, the onset of AD symptoms in persons with a heightened risk for AD.

## Supplementary information

**Additional file 1.** PAAD-2 SPIRIT Checklist

**Additional file 2.** PAAD-2 Cognitive Test Protocols

**Additional file 3.** PAAD-2 Data Safety Monitoring Plan

**Additional file 4.** PAAD-2 Data Sharing Plan

## Data Availability

Data sharing is not applicable to this article as no datasets were generated or analyzed during the current study which describes an on-going study protocol. Pre-randomization data will be made available within 12 months of enrollment completion. Post-randomization data will be made available upon publication of the main findings of the study or 2 years following study closure (whichever comes first). After these dates, the datasets used and/or analyzed will be available from the corresponding author on reasonable request.

## Abbreviations

ACSM: American College of Sports Medicine
AD: Alzheimer’s disease
*APOE4*: Apolipoprotein epsilon 4 allele
*APOE4*-: Non-carriers of the apolipoprotein epsilon 4 allele
*APOE4+*: Carriers of the apolipoprotein epsilon 4 allele
BDNF: Brain-derived neurotrophic factor
BOLD: Blood oxygenation level-dependent
DNA: Deoxyribonucleic acid
EF: Executive function
EPI: Echo planar imaging
FH: Family history of Alzheimer’s disease
FNDC5: Fibronectin type III domain containing 5
HR: Heart Rate
IGNITE: Investigating Gains in Neurocognition in an Intervention Trial of Exercise
LGC: Latent growth curve
MCI: Mild cognitive impairment
MoCA: Montreal Cognitive Assessment
MRI: Magnetic resonance imaging
MST: Mnemonic Similarity Task
NIH: National Institutes of Health
PA: Physical activity
PAC: Physical activity condition
PAR-Q: Physical Activity Readiness Questionnaire for Everyone
PSMT: Picture Sequence Memory Test
RAVLT: Rey Auditory Verbal Learning Test
RCT: Randomized control trial
RPE: Ratings of perceived exertion
SAP: Serum amyloid P
SNP: Single nucleotide polymorphism
SOP: Standard Operating Procedures
TICS-m: Modified Telephone Interview for Cognitive Status
UCC: Usual-care control
VCAP: Virginia Cognitive Aging Project
