# Supplementary material for: The effect of physical activity on cognition relative to APOE genotype (PAAD-2): study protocol for a phase II randomized control trial
Source: BMC Neurol. 2020 Jun 6;20:231. doi: 10.1186/s12883-020-01732-1 (PMC7274941; doi:10.1186/s12883-020-01732-1)
Supplement: Supplementary file 2 — Additional file 2. PAAD-2 Cognitive Test Protocols [file 12883_2020_1732_MOESM2_ESM.pdf]

PAAD2 Cognitive Test Protocol

Table S1. Timeline and order of PAAD-2 cognitive test battery

Set	Testing Tool	Time (minute)	Instrument
A	Session Introduction	2	
	Test of Premorbid Functioning (pre-test only) [a]	5	Paper
	NIH Toolbox Touchscreen Tutorial	2	iPad
	NIH Toolbox List Sort Working Memory Test	10	iPad
	NIH Toolbox Picture Sequence Test +	5	iPad
	Mnemonic Similarity Task +	10	Computer
	Perceptual Discrimination Task +	5	Computer
	*Break	5	
	Rey-Osterrieth Complex Figure Test; Copy Trial	2	Paper / Pencil
	*Break	3	
	Rey-Osterrieth Complex Figure Test; Immediate Recall Trial	2	Paper / Pencil
	Stroop Color-Word Task	10	Computer
	Paced Auditory Serial Addition Test - 3' and 2' Trials [a]	8 - 10	Computer
	*Break	5 - 7	
	Rey-Osterrieth Complex Figure Test; 30-min Delayed Recall & Recognition	5	Paper / Pencil
	Tower of London - Freiburg Version +	11	Computer
	*Break	5 - 10	
B	Rey Auditory Verbal Learning Test; Learning & Immediate Recall Trials [a] +	10	Computer
	NIH Toolbox Dimensional Change Card Sort Test	6	iPad
	NIH Toolbox Flanker Inhibitory Control and Attention Test	4	iPad
	Spatial Working Memory Task	10	Computer
	Trail Making Test; Part A & B	5	Paper / Pencil
	*Break	5	
	Rey Auditory Verbal Learning Test; 30-min Delayed Recall [a] & Recognition	3	Computer
	Paired Associates; Immediate Recall +	5	Computer
	Matrix Reasoning +	12	Computer
	Digits Span Forward & Backward Test	8	Computer
	Paired Associates; 20-min Delayed Recall +	2	Computer
	*Break	5	
	Logical Memory; Immediate Recall [a] +	10	Computer
	Spatial Relation +	12	Computer
	Symbol Digit Modalities Test; Written, Oral, and Incidental Learning	8	Paper / Pencil
	Logical Memory; 20-min Delayed Recall [a] +	3	Computer

Note. All testing tools are administered in the same order at pre-, mid-, and post-tests except for the Test of Premorbid Functioning (pre-test only). Total administration time is about 199 minutes (5 minutes shorter at mid- and post-tests). Set A and B are separately administered on two visits when needed. Administration time of Set A and B would be about 81 and 108 minutes, respectively.

* Breaks are provided for 5-10 minutes between every 20 – 35 minutes for participants to relax, eat, drink, or use the restroom.

[a] Participants' responses are audio-recorded along with manual records on standard forms.

+ Three different forms are used across repeated sessions at pre-, mid-, and post-tests.

Episodic memory test protocols

The Rey Auditory Verbal Learning Test (RAVLT) requires participants to immediately learn and recall a list of 15 unrelated words repeated over five different trials. Each word is spoken through a desktop computer at the rate of one word per second and 30 seconds are given for the recall phase (list A learning trials). Then, another list of 15 unrelated words is given and recalled (list B), and list A is recalled once again (immediate recall) and then again after 30 minutes (delayed recall), with a 1-minute time limit for both recall trials. Then, participants complete a recognition trial to identify all the words from the first and second lists from a list of 50 words, which includes 20 novel words. Based on meta-norms accumulated from previous studies (1), age-stratified normative scores are created for all RAVLT variables. All responses are recorded on a scoring form and audio-recorded at the same time. The test is administered via PsychoPy 3.0 (2) on a desktop computer.

The Virginia Cognitive Aging Project (VCAP; 3)'s version of the Paired Associates test (4) requires the participant to listen to pairs of words. Following the exposure period, the participant is immediately asked to recall the paired response word when presented with its paired stimulus word once. Participants perform two trials consisting of exposure and immediate recall of 6 pairs of words. Then, 20-minute later, participants are asked to say the paired response word again in response to its paired stimulus word without hearing the pairs again (delayed recall). All word stimuli are presented by E-Prime 3.0 (5) on a desktop computer at the rate of one word per second. During the immediate recall phase, 3 seconds are given for answering each response word and a prompt is shown on the screen for the next word. During the delayed recall phase, the staff member says each stimulus word in a random array of all 12 words, and participants are required to say the paired response word with no time limit (max score 12 points). All responses are recorded and scored on a paper form.

The VCAP's version of the Logical Memory test (6) requires the participant to recall specific idea units from two stories consisting of 6-7 sentences immediately after listening once (immediate recall). After immediate recall for each story (trial 1 and 2), learning and recalling of the second story is repeated (trial 3). Twenty minutes later, participants are asked to verbally recall each story again (delayed recall). Two stories are provided in each of three testing sessions. The duration of each of six stories are 24 – 38 seconds. All stories have 25 idea units (max score 25 points). Stories are presented by E-Prime 3.0 (5) on a desktop computer. Participants' responses are recorded on a scoring form and audio-recorded at the same time. Scores on the paper form are double-checked with the audio recording by a staff member.

The Digits Span Forward Test (7) is a test of immediate recall of a series of single digit numbers presented visually. Specifically, the task requires participants to immediately say back the digit items in the order presented. The Digits Span Backward Test (7) is administered in the same fashion as the Digits Span Forward task, but requires participants to immediately say back the digit items in the reverse order. The number of digits in the list starts at 3 and increases up to 9 with two trials for each list length (14 trials in total). While the Digits Span Forward task measures short-term verbal memory as it requires simply holding information in mind, the Digits Span Backward task provides a measure on working memory as it requires the rearrangement of

the digits into backward order (8). The digit items are presented via E-prime 3.0 on a desktop computer at a rate of one digit per second and participants' responses are recorded on the computer. The total number of trials and the longest span of digits correctly recalled are calculated. Based on demographics of a normative standard sample, age-, gender-, and education-adjusted scores are created (7, 9).

The Rey-Osterrieth Complex Figure Test (ROCFT) requires participants to accurately draw the figure shown on the ROCFT stimulus card (copy trial). After 3 minutes of filler instructions and conversation regarding study participation and the upcoming schedule, participants are asked to draw the same figure without the stimulus card (immediate recall). Thirty minutes later participants are asked to draw the figure again (delayed recall). Then, participants complete a recognition trial to identify 12 partial segments of the entire figure from a list of 24 partial figures. Drawings from each trial are scored based on the accuracy, placement, and organization of 18 scoring units. Each unit is given points from 0, 0.5, 1, or 2, so the total score for each drawing trial is 0 – 36. Recognition total correct score is the sum of the recognition true positive and true negative scores ranging from 0 to 24. Raw scores are transformed into age-stratified scores based on normative data (10).

The National Institutes of Health (NIH) Toolbox Picture Sequence Memory Test requires participants to reproduce the order of a randomized sequence of 15 - 18 pictures of thematically associated objects and activities presented on an iPad screen. The order of the objects and activities presented has no inherent constraints, yet participants are required to remember and reproduce the specific order in which the pictures are presented. Verbal instructions and practice trials ensure that participants learn the requirements of the test. Each picture is presented for 2.2 seconds, then reduces in size and moves to its position in the sequence for 1.5 seconds and remains until all pictures are presented. Three seconds after the entire sequence is shown, the pictures are mixed up in a random spatial array at the center of the screen. Then, participants are required to drag each picture to its correct place on the screen, with no time limit. Two trials of the same sequence are administered to improve test score variability and test–retest reliability. Points are given to each adjacent pair of pictures correctly placed. The maximum score is the number of adjacent pairs of pictures, which is one less than the sequence length. Trial 1 and 2 have 15 and 18 pictures, respectively, so a maximum of 31 points are given at the end. Based on demographics of a normative standard sample, age-, gender-, race-, ethnicity-, and education-adjusted scores are created for all tests in the NIH Toolbox cognition battery (9).

The Mnemonic Similarity Task (MST; 11) is a behavioral episodic memory task designed to assess recognition memory and mnemonic discrimination for pictures of everyday objects (e.g., a picnic basket). Importantly, performance on specific measures from this task are thought to assay behavioral pattern separation, which is a computational mechanism that facilitates the encoding of overlapping information as distinct inputs and reduces interference among similar representations (e.g., encoding and representing two similar picnic baskets as being the same type of object but different objects). This task comprises study and test phases in which participants first view a list of everyday objects and then are asked to identify which objects are repetitions of earlier objects (studied), have the same identity as studied objects but vary on some

perceptual features (similar), and did not appear earlier (unstudied). Pattern separation is operationalized as accuracy in identifying similar objects as such minus bias to classify unstudied objects as similar. Based on a set of previously validated stimuli (11), we developed and validated a custom version of the task. Images are presented via E-prime 3.0 on a desktop computer. For each object, participants are asked to determine if it is an indoor or an outdoor object. After viewing 72 objects, participants are shown 36 of these objects, 36 similar objects, and 36 unstudied new objects and are asked to distinguish whether each image is "Old", "Similar", or "New", respectively.

The Perceptual Discrimination Task (PDT) follows immediately after the MST. The PDT examines participants' ability to distinguish between similar objects when they appear on the screen simultaneously. This measure is used as a covariate to control for differences in perceptual discrimination when assessing differences in pattern separation in the MST. The PDT consists of the same everyday objects as the MST. Pairs of objects appear individually, and participants are instructed to indicate whether those pairs are the "Same", "Similar", or "Different." Same pairs consist of the same studied object from the MST; Similar pairs consist of the two different versions of the same object from the MST; and Different pairs consist of two different objects that were new objects on the test phase of the MST. The PDT includes 36 same, 36 similar, and 18 different pairs.

Core executive function test protocols

We use the same version of the Stroop color-word task as is used in a similar clinical trial (Investigating Gains in Neurocognition in an Intervention Trial of Exercise, IGNITE trial; 12). The task is administered via E-Prime 3.0 (5) on a desktop computer. A series of colored words (red, blue, or green) are randomly presented in congruent colors (i.e., the word 'green' displayed in green ink) and incongruent colors (e.g. the word 'green' displayed in either red or blue ink). Subjects are instructed to indicate what color each word stimulus is printed in, regardless of the semantic information of the word, as quickly and as accurately as possible using the "B", "N", and "M" keys on a keyboard. Nine practice trials with feedback are provided before test trials to acquaint participants with the task instructions. A total of 114 test trials are presented (36 congruent, 36 incongruent, and 42 neutral trials) in a randomized order based on a list of 30 stimuli consisting of 3 congruent (red, blue, and green in congruent colors), 6 incongruent (red, blue, green in incongruent colors), and 21 neutral stimuli (ship, home, chair, road, door, dog, and jump in red, blue, and green colors). Responses must be given within a 2.5s time limit. When more than half of the practice trials are missed, the practice block is repeated up to two more times. Accuracy and reaction time on the incongruent trials serve as measures of inhibitory control whereas accuracy and reaction time on the congruent and neutral trials provide measures of visual selective attention and processing speed.

As a reversed form of the Digit Symbol Substitution Test (DSST; 13), the Symbol Digit Modalities Test (SDMT) was developed to identify individuals with neurological impairment by assessing attentional control, visual search, and visuomotor coordination (14). The SDMT requires participants to pair nine geometric figures with numerical digits from 1 to 9 based on a

reference key. Participants must write (manual trial, trial 1) or verbally say (oral trial, trial 2) the correct number for each randomly generated geometric figure on a paper sheet. Participants are given 10 practice items before beginning the test trials. The final score is the correct number matched for the figures within a 90-second time limit for each trial (maximum 110). After two trials, participants are asked to recall 15 numbers paired with each digit without a reference key (maximum point 15) (incidental learning trial). The incidental learning trial provides a measure of visual episodic memory/learning (15). We calculate age- and education-stratified scores based on normative data (14, 16).

The Paced Auditory Serial Addition Test is a measure of attentional control, auditory information processing speed, and calculation ability. We use 3- and 2-second inter-stimulus interval (ISI). For the test administration, we use a desktop computer to present a series of 61 single-digit numbers either every 3 seconds (trial 1) or every 2 seconds (trial 2). While listening to the digits, participants must add each new digit to the one presented immediately prior to it. For example, if the digits '3', '5' and '7' are presented, participants must respond with the correct sums, '8' (= 3 + 5) and '12' (= 5 + 7). Responses are scored as correct when responded during the ISI after the presentation of the digit or in the next interval given no response is given in the preceding interval (17). Based on demographics of national normative data (16, 17), age- and education-adjusted scores are created. The test is administered via PsychoPy 3.0 (2) on a desktop computer.

The NIH Toolbox Flanker Inhibitory Control and Attention test requires participants to indicate the left–right orientation of an arrow stimulus presented centrally while inhibiting attention to the potentially incongruent arrow stimuli surrounding the central stimulus. Four practice trials with feedback are given to acquaint participants with test instruction, then a total of 20 test trials are presented. When participants miss more than one of the practice trials, 4 practice trials are repeated up to two more times. In the test trials, 12 trials have the orientation of the surrounding stimuli congruent with the orientation of the central stimulus, and it is incongruent on the other 8 trials. The congruent and incongruent trials are presented in pseudorandom order, with 1–3 congruent trials preceding each incongruent trial. The word "middle" is visually presented in order to remind participants to attend to the central stimulus. Participants are required to respond as quickly and accurately as possible by touching the response options on the iPad screen. Accuracy and reaction time on the incongruent trials serve as measures of inhibitory control in the context of visual selective attention, which can also be a measure of executive attention (18).

The NIH Toolbox List Sorting Working Memory Test requires participants to remember, sort, and sequence visual and auditory information presented on an iPad. A series of illustrated pictures, each depicting an item (e.g., an animal or a fruit), are displayed for 2 seconds, along with the auditory information of their names. Participants must remember the stimuli to verbally repeat them to the administrator in size order, from smallest to biggest. The span of stimuli, the number of objects in a series, increases on succeeding items from 2 to 7 objects, consequently taxing the working memory system when longer span needs to be remembered (19). The first block of the test (list 1) requires participants to arrange one type of stimuli (e.g., "animals" or

“food”) in order by size. Then the second block of the test (list 2) requires sorting out both food and animal stimuli, each in size order, which substantially increases working memory load through “dual” tracking and processing systems as participants have to remember and organize stimuli from both categories and report in size order for each category (19). Two practice items for each block are provided for participants to learn the test instructions for each block.

When performing the NIH Toolbox Dimensional Change Card Sort Test, participants first see two reference pictures (e.g., white rabbit and brown boat) and then a test picture that could differ in one of two dimensions [e.g., rabbit or boat (shape), white or brown (color)]. Participants then must match a series of test pictures to the reference pictures based on one dimension (e.g., shape). They must learn which dimension is being matched based upon trial and error and the feedback they receive. After correctly identifying the matching dimension, the sorting dimension changes unbeknownst to the participant. Four practice trials are given for each dimension to acquaint participants with test instructions. When participants miss more than one of the practice trials, 4 practice trials are repeated up to two more times. Then, 30 test trials (e.g., ball or truck in yellow or blue) are required to be completed in the same manner. Participants are required to respond as quickly and accurately as possible by touching the response picture on the screen. Participants’ performance is scored based on a two-vector method to create a composite score that reflects first accuracy and then, reaction time.

The IGNITE’s spatial working memory task (20) is administered via E-prime 3.0 (5) on a desktop computer to measure visuospatial working memory. At the beginning of the test, a fixation cross appears for 1 second, and participants are required to keep their eyes on the cross. After the fixation, one, two, or three black dots appear in random locations on the screen for 500 ms. Then, the dots are removed from the screen for 3 seconds, and participants are required to remember the locations of the black dots previously presented. After 3 seconds, a red dot appears on the screen either at the same location as one of black dots (matching condition) or at a different location (non-matching condition). Within a 2-second time limit, participants have to respond to the red dot as quickly and accurately as possible by pressing one of two keys on a keyboard; the “x” for a non-matching condition and the “m” for a matching condition. Six practice trials with feedback are provided before test trials to acquaint participants with the task instructions. When more than half of the practice trials are missed, the practice block is repeated up to two more times. After practice trials, a total of 40 test trials (20 matching and 20 non-matching conditions) are presented for each set of one, two, and three dots.

We use part A and B within the Trail Making Test (21, 22). Part A requires the participant to sequentially draw lines through 25 numbered circles which are randomly distributed on a sheet of paper. Part B requires participants to connect 25 encircled numbers and letters in numerical and alphabetical order, alternating between the numbers and letters (e.g., 1, A, 2, B, 3, C, etc.). The numbers and letters are placed in a semi-randomized order to avoid overlapping lines while drawing. It is generally presumed that part A measures visual search and motor speed skills and part B measures higher level cognitive skills such as cognitive flexibility (22-24). The primary variables of interest are the total time to complete each of part A and B. A time limit of 300 seconds is used to discontinue each trial and is therefore the maximum score.

Errors during trials do not directly count but indirectly contribute to the total time to complete the test as the participants have to be stopped and returned to the last correct response (23). Raw scores are transformed into age-stratified scores based on normative data (21).

Higher-order executive function test protocols

The Tower of London – Freiburg version (TOL-F) is operated based on a realistic three-dimensional representation of Shallice (25)'s original wooden model of the tower configuration administered with the Vienna Test System (VTS; SCHUHFRIED GmbH) on a desktop computer. The TOL-F consists of three rods of different heights on which three colored balls (red, yellow, and blue) are placed. The left, middle, and right rods can hold three, two and one ball(s), respectively. For each problem, the goal state and the start state are presented in the upper and lower parts of the screen, respectively. To solve a problem successfully, participants must modify the start state to be the same as the goal state in the minimum number of moves, which is shown on the left next to the start state. Using a computer mouse, participants have to move balls one by one between rods in the start state configuration. Balls blocked by other balls lying on top of them cannot be moved and rods already filled with balls in full cannot hold more balls. The VST records the number of attempts to break these rules when moving balls. A progress bar of the one-minute time limit is shown in the upper-right corner when working on each problem. The problem being worked on is automatically discontinued when the time limit is exceeded. If the time limit is exceeded for three consecutive problems, the TOL-F is automatically terminated.

We use the short form of the TOL-F that consists of 14 problems (two three-move, two four-move, five five-move, and five six-move problems) selected from 28 problems in the standard form based on their high correlation with the standard form, high reliability, high sensitivity, and acceptable specificity (26). Participants' performance is automatically recorded, and an age-stratified percentile is calculated based on raw scores. The raw planning score (12 points in total) is counted based on the total number of problems solved in the minimum number of four-, five-, and six-moves.

The VCAP's version of the Matrix Reasoning test (27) requires the participant to determine the best fitting piece of a pattern that completes the missing cell in a 3x3 matrix. Participants are given test instructions and complete two practice trials. When participants miss both practice trials, the instruction is repeated, and the correct solution is explained. Then, test trials require participants to work on 18 problems within a 10-minute time limit. The Matrix Reasoning test is presented via a custom-built program in E-Prime 3.0 (5) on a desktop computer. Participants' responses are automatically recorded, and accuracy and reaction time are calculated through the program.

The VCAP's version of the Spatial Relation test (28) requires the participant to determine the exact match between a three-dimensional figure and a two-dimensional figure. Each problem provides a two-dimensional figure on the upper half of the screen. Participants are to consider the two-dimensional figure to be like a piece of paper that can be folded. They are to identify which of four choices of three-dimensional figures presented at the lower half of the screen could be

created from the two-dimensional figure. Participants are given instructions for the test along with two example problems. The instruction and the solution of the example problems are repeated when participants don't understand. Then, test trials allow the participant to work on as many problems as possible from a set of 20 problems within a 10-minute time limit. The Spatial Relation test is presented via a custom-built program in E-Prime 3.0 (5) on a desktop computer. Participants' responses are automatically recorded, and accuracy and reaction time are calculated through the program.

Abbreviations

DSST: Digit Symbol Substitution Test; IGNITE: Investigating Gains in Neurocognition in an Intervention Trial of Exercise; MST: Mnemonic Similarity Task; NIH: National Institute of Health; PDT: Perceptual Discrimination Task; RAVLT: Rey Auditory Verbal Learning Test; ROCFT: Rey-Osterrieth Complex Figure Test; SDMT: Symbol Digit Modalities Test; TOL-F: Tower of London – Freiburg version; TOPF: Test of Premorbid Functioning; VCAP: Virginia Cognitive Aging Project; VTS: Vienna Test System

References

1. Schmidt M. Rey Auditory Verbal Learning Test: A Handbook. Los Angeles, CA: Western Psychological Services; 2004.
2. Peirce J, Gray JR, Simpson S, MacAskill M, Höchenberger R, Sogo H, et al. PsychoPy2: Experiments in behavior made easy. Behavior Research Methods. 2019;51(1):195-203.
3. Salthouse TA. Contributions of the Individual Differences Approach to Cognitive Aging. J Gerontol B Psychol Sci Soc Sci. 2017;72(1):7-15.
4. Salthouse TA, Fristoe N, Rhee SH. How localized are age-related effects on neuropsychological measures? Neuropsychology. 1996;10(2):272-85.
5. Psychology Software Tools I. E-Prime 3.0. Pittsburgh, PA: Retrieved from https://www.pstnet.com; 2016.
6. Wechsler D. Wechsler memory scale. 3rd ed. San Antonio, TX, US: The Psychological Corporation; 1997.
7. Stern RA, White T. Neuropsychological Assessment Battery (NAB) Digits Forward/Digits Backward Test Professional Manual. Lutz, FL: Psychological Assessment Resources; 2003.
8. Diamond A. Executive functions. Annual review of psychology. 2013;64:135-68.
9. Casaletto KB, Umlauf A, Beaumont J, Gershon R, Slotkin J, Akshoomoff N, et al. Demographically Corrected Normative Standards for the English Version of the NIH Toolbox Cognition Battery. J Int Neuropsychol Soc. 2015;21(5):378-91.
10. Meyers JE, Meyers, Kelly R. Rey Complex Figure Test and Recognition Trial Professional Manual. Lutz, Florida: PAR; 1995.
11. Stark SM, Yassa MA, Lacy JW, Stark CE. A task to assess behavioral pattern separation (BPS) in humans: Data from healthy aging and mild cognitive impairment. Neuropsychologia. 2013;51(12):2442-9.
12. Erickson KI, Grove GA, Burns JM, Hillman CH, Kramer AF, McAuley E, et al. Investigating Gains in Neurocognition in an Intervention Trial of Exercise (IGNITE): Protocol. Contemporary clinical trials. 2019;85:105832.
13. Wechsler D. WAIS-R : Wechsler adult intelligence scale-revised. New York, N.Y. : Psychological Corporation, [1981] ©1981; 1981.
14. Smith A. Symbol Digit Modalities Test Manual (W-129C). Torrance, CA: Western Psychological Services; 2011.

15. Denney DR, Hughes AJ, Elliott JK, Roth AK, Lynch SG. Incidental learning during rapid information processing on the symbol-digit modalities test. Archives of clinical neuropsychology : the official journal of the National Academy of Neuropsychologists. 2015;30(4):322-8.
16. Kiely KM, Butterworth P, Watson N, Wooden M. The Symbol Digit Modalities Test: Normative data from a large nationally representative sample of Australians. Archives of clinical neuropsychology : the official journal of the National Academy of Neuropsychologists. 2014;29(8):767-75.
17. Tombaugh TN, Rees L, Baird B, Kost J. The Effects of List Difficulty and Modality of Presentation on a Computerized Version of the Paced Serial Addition Test (PSAT). Journal of Clinical and Experimental Neuropsychology. 2004;26(2):257-65.
18. Zelazo PD, Anderson JE, Richler J, Wallner-Allen K, Beaumont JL, Weintraub S. II. NIH Toolbox Cognition Battery (CB): measuring executive function and attention. Monographs of the Society for Research in Child Development. 2013;78(4):16-33.
19. Tulsky DS, Carlozzi NE, Chevalier N, Espy KA, Beaumont JL, Mungas D. V. NIH TOOLBOX COGNITION BATTERY (CB): MEASURING WORKING MEMORY. Monographs of the Society for Research in Child Development. 2013;78(4):70-87.
20. Erickson KI, Voss MW, Prakash RS, Basak C, Szabo A, Chaddock L, et al. Exercise training increases size of hippocampus and improves memory. Proceedings of the National Academy of Sciences. 2011;108(7):3017-22.
21. Reynolds CR. Comprehensive Trail-Making Test Examiner's Manual. Austin, Texas: PRO-ED, Inc.; 2002.
22. Crowe SF. The differential contribution of mental tracking, cognitive flexibility, visual search, and motor speed to performance on parts A and B of the Trail Making Test. J Clin Psychol. 1998;54(5):585-91.
23. Bowie CR, Harvey PD. Administration and interpretation of the Trail Making Test. Nature Protocols. 2006;1(5):2277-81.
24. Kortte KB, Horner MD, Windham WK. The trail making test, part B: cognitive flexibility or ability to maintain set? Appl Neuropsychol. 2002;9(2):106-9.
25. Shallice T. Specific impairments of planning. Philos Trans R Soc Lond B Biol Sci. 1982;298(1089):199-209.
26. Kaller CP, Unterrainer JM, Kaiser S, Weisbrod M, Debelak R, Aschenbrenner S. VIENNA TEST SYSTEM MANUAL. TOWER OF LONDON - FREIBURG VERSION. Mödling, Austria: SCHUHFRIED GmbH; 2011.
27. Raven J. Advanced progressive matrices, set II. London: H. K. Lewis; 1962.
28. Bennett GK, Seashore HG, Wesman AG. Differential Aptitude Tests. San Antonio, TX: Psychological Corporation; 1997.
