# Supplementary material for: The effect of physical activity on cognition relative to APOE genotype (PAAD-2): study protocol for a phase II randomized control trial
Source: BMC Neurol. 2020 Jun 6;20:231. doi: 10.1186/s12883-020-01732-1 (PMC7274941; doi:10.1186/s12883-020-01732-1)
Supplement: Supplementary file 3 — Additional file 3. PAAD-2 Data Safety Monitoring Plan [file 12883_2020_1732_MOESM3_ESM.pdf]

Data Safety Monitoring Plan

The effect of physical activity on cognition relative to APOE genotype (PAAD-II): Study protocol for a phase II randomized control trial

R01 AG058919

Jennifer L. Etnier
University of North Carolina at Greensboro

TABLE OF CONTENTS

Page

1.0 Participants' Safety 1

1.1 Potential Risks and Benefits for Participants 1

1.2 Adverse Event and Serious Adverse Event Collection and Reporting 1

1.3 Protection Against Study Risks 2

2.0 Data and Safety monitoring 3

2.1 Frequency of Data and Safety Monitoring 4

2.2 Content of Data and Safety Monitoring Report 4

2.3 Conflict of Interest for Safety Officer 4

2.4 Protection of Confidentiality 4

2.5 Safety Officer Role, Responsibilities, and Procedures 4

1.0 PARTICIPANTS' SAFETY

1.1 Potential Risks and Benefits for Participants

Potential Risks: The potential risks to study participants come from 5 sources.

1. *Exclusion criteria:* There is a risk of a negative emotional response if participants are excluded because they meet criteria for clinical cognitive impairment or meet the criteria for depression based upon depressive symptoms. Similarly, if participants are told that they must obtain physician clearance to exercise because of their health, they may have a negative emotional response.

2. *Exercise:* Potential risks include muscle fatigue, soreness, and dizziness during and after the exercise and falling during exercise or when walking to or from sessions. Abnormal changes in heart function, and, in very rare instances, heart attack (non-fatal or fatal) may also occur during exercise.

3. *Genotype information:* There is a risk of a negative emotional response if participants were to become aware of their genotyping information.

4. *Blood samples:* Slight discomfort and/or infection is possible when blood samples are taken. Bruising and mild-to-moderate soreness to the touch may occur at the site following the blood draw.

5. *MRI:* During the MRI, there is the risk of exposure to loud noise, the heating of metal in the body, an experience of claustrophobia, uncomfortable muscle twitching or tingling, and experiences of dizziness, taste sensations, or light flashes. Although this is not a clinical scan and it will not be reviewed by medical staff, it is possible that the researchers might notice substantial deviations from normal anatomy when viewing the images. If this happens, we will contact participants to let them know this, and they may experience a negative emotional response.

Potential Benefits: The potential benefits to study participants include possible physical and mental health benefits for participants in response to the exercise program.

1.2 Adverse Event and Serious Adverse Event Collection and Reporting

For each adverse or unanticipated event serious and non-serious, the research staff will use the PAAD-2 Unanticipated Problem/Adverse Event (AE) Form to provide the PI (Etnier) with information regarding the location, research personnel present, onset, duration, treatment, action taken, and outcome. Forms for such reporting will be available at all data collection sessions and during the physical activity program.

Research staff will be instructed that all events should be reported immediately to the PI, but at least within 24 hours. In the event that the PI is not available, the research staff will report directly to the Co-Investigators on the grant (Karper, Wideman). Deaths will be reported to the UNCG Office of Research Integrity (ORI), the Safety Officers (Williamson, Cleveland), and the NIA Program Officer (McLinden) within 24 hours of study's knowledge by the PI. Unanticipated SAEs will be reported to the Safety Officers and the NIA Program Officer within 48 hours of study's knowledge by the PI. Summaries of AEs and all other SAEs will be reported to the NIA Program Officer bi-annually by the Safety Officers.

With regards to training of research staff, those who are working with participants will be instructed and regularly reminded that any adverse or unexpected event that is reported to or observed by the study staff should be documented and reported. In addition, the PI will observe a minimum of 5% of the pre-test submaximal fitness assessments and Dr. Bill Karper will train exercise specialists and regularly observe exercise sessions to ensure that the research staff is compliant with the research protocol.

Necessary follow-up will be dependent upon the identification of the event as an AE or an SAE and whether the event was expected or unexpected and related, possibly related, or unrelated to the study protocol. All staff will be made aware of any necessary modifications to protocol.

1.3 Protection Against Study Risks

Informed Consent Process. The consent process informs a volunteer about the study, indicates that participation is voluntary, and lets the participant know that he/she has the right to stop at any time. Risks are enumerated in the informed consent form and described orally during the consent process.

Protection Against Risks. All study personnel will participate in Good Clinical Practice (GCP) and Responsible Conduct of Research (RCR) education programs. In addition, the following protections will be taken relative to the previously identified sources of risk.

1. *Exclusion criteria:* Dr. Tomika Williams (Nurse Practitioner) will contact participants excluded for cognitive impairment or depression. Dr. Tomika Wiliams's contact information will be made available to participants who are required to get physician approval to exercise. Dr. Williams will answer any questions that they may have and encourage them to schedule an appointment with their personal physician if they want to follow-up.

2. *Exercise:* Participants will be asked to complete a medical health history questionnaire that will be used in conjunction with the American College of Sports Medicine guidelines (10th Edition, 2018) to make a determination of risk for participation in regular exercise and for completing the submaximal exercise test. The risk categories are (1) apparently healthy, (2) having known disease and asymptomatic, or (3) symptomatic. Those who are apparently healthy can safely participate in moderate-to-vigorous intensity exercise (as will be used in this intervention and in the exercise testing) according to the ACSM guidelines. Although not required for study inclusion, these individuals will be asked to provide physician consent prior to participation. Those who have known disease and are asymptomatic and those who are symptomatic must provide evidence of medical clearance for participation, but then may participate in moderate intensity exercise. Those who have known disease that is poorly managed (i.e., they are symptomatic and unstable) will not be allowed to participate.

   To reduce risk from exercise, we will use experienced exercise staff who are familiar with the warning signs of heart attack and stroke, who are trained in cardiopulmonary resuscitation (CPR), and who know procedures to contact emergency personnel. To reduce the risk of falls, we will ask participants to exercise at a self-selected pace during the exercise program and will use handrails on the treadmill for the submaximal exercise test. Emergency personnel will be contacted right away in the event of a life-threatening

emergency. Participants will be required to wear proper shoes while exercising. Exercise will be at a moderate intensity and progressed at an individual level as tolerated. Aerobic exercise will be monitored using ratings of perceived exertion and heart rate as assessed by palpation to ensure that exercise remains at a moderate level. Heart rate at rest and during exercise will be assessed every 3 weeks using a heart rate monitor so that the correct target heart rate can be identified. Strength training resistance, reps, and sets will be progressed based upon each individual's tolerance.

Participants in the physical activity condition who sustain any type of injury or who experience cardiovascular symptoms (inside or outside of the program) will be asked to notify the Exercise Specialist who will make appropriate recommendations based upon ACSM guidelines. This may include modifying or stopping exercise for that individual for a period of time or requiring permission from a physician to resume exercise. At the mid-test and post-test, participants in both groups will be asked to complete a standardized questionnaire assessing any adverse events experienced since the last visit.

3. *Genotype information:* To protect against risks of finding out their genotype, this information will be stored in a data file that only includes ID#. Research staff regularly interacting with participants will not have access to the master list linking names to ID#s.

4. *Blood samples:* To protect against risks from the blood draws, experienced staff trained in phlebotomy will conduct the blood draws using sterile techniques. OSHA techniques will be followed for blood draws and handling of biological samples. Participants will be instructed in how to use ice to reduce bruising and soreness.

5. *MRI:* To protect against risks from the MRI, screening for safety and MRI scans will be performed. Participants will be asked a series of questions about their medical history to determine if an MRI exam is safe (in advance of their scheduled scan) and will be asked these same questions on the day of their scan. Their responses will be reviewed by a Level 4 trained MRI operator. During the scan, participants will hold a squeeze ball that they can use to indicate if they need to come of out the scanner at any time and, upon squeezing the ball, they will be removed as quickly as possible. They will also wear MRI-specific ear plugs to reduce noise. On the consent form, participants will be told that the research team cannot diagnose conditions. But, they will also be told that if the research team sees a substantial deviation from normal anatomy, the MRI post-doctoral fellow will contact them to explain the abnormality, will offer Dr. Tomika Williams' (Nurse Practitioner) contact information for follow-up questions, will provide them with a free copy of their data, and will suggest that they contact their physician for follow up.

2.0 DATA AND SAFETY MONITORING

The Principal Investigator (PI) will be responsible for ensuring that research staff have the necessary training and education as per the DSMP. The Safety Officers will act in an advisory capacity to the NIA to monitor participant safety, evaluate the progress of the study, and to review procedures for maintaining the confidentiality of data, the quality of data collection, data management, and analyses.

2.1 Frequency of Data and Safety Monitoring

The PI with the help of the statistician and other research staff will prepare data and safety monitoring reports to be sent to the SO every 6 months following the initial meeting to approve the DSMP (11.27.2018). These reports will include a detailed analysis of study progress, data quality, and safety issues. Seven to 14 days following receipt of the report, the Safety Officer will meet with the PI either in-person or virtually to review study progress, data quality, and participants' safety and will then forward the report to the NIA Program Officer.

2.2 Content of Data and Safety Monitoring Report

The content of the data and safety monitoring report will include recruitment, compliance, and retention data, participant demographic information, and safety information. A formal report containing the data and safety monitoring report and recommendations for continuation or modifications of the study will be prepared by the Safety Officer and submitted to the NIA and the PI. It is the responsibility of the Principal Investigator to distribute the Safety Officer recommendations for modifications to all co-investigators and to ensure that copies are submitted to all the IRBs associated with the study.

2.3 Conflict of Interest for Safety Officer

The Safety Officer will have no direct involvement with the study investigators or intervention. The Safety Officer will sign a Conflict of Interest Statement which includes current affiliations, if any, with pharmaceutical and biotechnology companies (e.g., stockholder, consultant), and any other relationship that could be perceived as a conflict of interest related to the study and/or associated with commercial interests pertinent to study objectives.

2.4 Protection of Confidentiality

Initially, data presented to the Safety Officers in the bi-annual reports will be blinded such that summary data is only described as coming from Group A or Group B. If necessary to identify appropriate follow-up steps, data may be unblinded. The Safety Officer will sign a statement of confidentiality indicating that reports, data, and the content of discussions are confidential.

2.5 Safety Officer Responsibilities

The Safety Officer's Responsibilities are:

- Review the research protocol, informed consent documents and plans for data safety and monitoring;
- Recommend subject recruitment be initiated after receipt of a satisfactory protocol;
- Evaluate the progress of the trial, including semi-annual assessments of data quality and timeliness, recruitment, accrual and retention, participant risk versus benefit, performance of the trial sites, and other factors that can affect study outcome;
- Consider factors external to the study when relevant information becomes available, such as scientific or therapeutic developments that may have an impact on the safety of the participants or the ethics of the trial;

- Review study performance, make recommendations and assist in the resolution of problems reported by the Principal Investigator;
- Protect the safety of the study participants;
- Report to NIA on the safety and progress of the trial;
- Make recommendations to the NIA and the Principal Investigator concerning continuation, termination or other modifications of the trial based on the observed beneficial or adverse effects of the treatment under study;
- Review procedures being used to ensure the confidentiality of the study data and the results of monitoring; and,
- Assist the NIA by commenting on any problems with study conduct, enrollment, sample size, and/or data collection.
