# Supplementary material for: The effect of physical activity on cognition relative to APOE genotype (PAAD-2): study protocol for a phase II randomized control trial
Source: BMC Neurol. 2020 Jun 6;20:231. doi: 10.1186/s12883-020-01732-1 (PMC7274941; doi:10.1186/s12883-020-01732-1)
Supplement: Supplementary file 4 — Additional file 4. PAAD-2 Data Sharing Plan [file 12883_2020_1732_MOESM4_ESM.pdf]

DATA SHARING PLAN

The effect of physical activity on cognition relative to APOE genotype (PAAD-II)

R01 AG058919

Jennifer L. Etnier

University of North Carolina at Greensboro

We recognize the value of sharing data from PAAD-2 with the scientific community. We seek to disseminate our results through a variety of outlets and make our raw data available to the community of scientists interested in Alzheimer's disease to advance our understanding of this disease.

1. **Our plan for dissemination includes the following:**

   a) **Presentations at national scientific meetings.** From PAAD-2, it is expected that approximately fourteen presentations will be given at national or international meetings. In addition, papers will be presented at local or regional meetings as appropriate. The PI is a fellow of the National Academy of Kinesiology and the American College of Sport Medicine and an active member of the North American Society for the Psychology of Sport and Physical Activity. Members of these organizations are very interested in understanding the effects of exercise on cognitive performance and in potential mechanisms of the effects. The PI and members of the research team will also disseminate results at conferences focused on Alzheimer's disease (Alzheimer's Association International Conference, Alzheimer's Disease and Dementia Conference) and/or those focused on neurocognitive mechanisms (e.g., Cognitive Neuroscience, Neurology and Cognitive Neuroscience, Neuroscience).

   b) **Publications.** Data used to support presentations will also be considered for publication and where the data are sufficient, manuscript preparation will follow. We anticipate publishing our findings in tier-1 journals.

2. **Our plan for sharing of data includes the following:**

   a) **What data will be shared:**
   Data documentation and de-identified data to include demographic data, behavioral data, biomarker data, MRI data, and genotype information will be deposited for sharing consistent with applicable laws and regulations. Data documentation and data will be made available in a de-identified anonymous state and in a .csv format.

   b) **How the data will be shared:**
   Data associated with the project will be shared by exporting the data from RedCap into a .csv file that will be archived under my institutional profile with UNC Greensboro University Libraries institutional repository NC DOCKS. This is a searchable archives. I will also become a data partner with the Global Alzheimer's Association Interactive Network (GAAIN). GAAIN is a federated data system designed to foster data sharing and the development of collaborations for researchers interested in Alzheimer's related data. Through GAAIN, interested scientists can explore meta data from PAAD-2 and from other related studies to find potentially relevant data to answer their own research questions. By becoming a GAAIN partner, a description of PAAD-2 and a link to contact the PI will be made available at www.gaain.org. I will identify where the data will be available and how to access the data in publications and presentations that I author or co-author about these data.

   c) **Who will have access to the data:**
   Investigators interested in having access to the data will submit their request through GAAIN and then will be asked to submit a proposal to the PI. The proposal should include institutional affiliation, a current resume or vita, source of funding (if applicable), and a detailed explanation of the research question and the data required. All applicants will also be required to sign an agreement of confidentiality. This agreement prohibits the use of the data in any way that would allow for the identification of individual participants.

   d) **When the data will be shared:**
   Consistent with the recommendations from the Collaboration for Alzheimer's Prevention (CAP) (Weninger, Carrillo, Dunn, Aisen, et al., 2016), pre-randomization data will be deposited within 12 months of enrollment completion. Consistent with the NIH guidelines, post-randomization data will be embargoed until publication of the the main findings of the study (i.e. those findings relevant to the specific aims) or two years following study closure (whichever comes earlier). Requests for data sharing that come before the end of the embargo period will be considered on a case-by-case basis by the PI.
